# Irreversible Electroporation of the Hepatobiliary System: Current Utilization and Future Avenues

**DOI:** 10.3390/medicina60020251

**Published:** 2024-01-31

**Authors:** Govindarajan Narayanan, Yilun Koethe, Nicole Gentile

**Affiliations:** 1 Miami Cancer Institute, Baptist Health South Florida, Miami, FL 33176, USA; nicole.gentile@baptisthealth.net; 2 Miami Cardiac and Vascular, Baptist Health South Florida, 8900 North Kendall Drive, Miami, FL 33176, USA; 3 Herbert Wertheim College of Medicine, Florida International University, Miami, FL 33199, USA; 4 TRG Imaging, Portland, OR 97225, USA; yilun.koethe@gmail.com

**Keywords:** liver cancer, irreversible electroporation, hepatocellular carcinoma, liver metastases, cholangiocarcinoma

## Abstract

Liver cancer remains a leading cause of cancer-related deaths worldwide despite numerous advances in treatment. While surgical resection remains the gold standard for curative treatment, it is only possible for a minority of patients. Thermal ablation is an effective option for the treatment of smaller tumors; however, its use is limited to tumors that are not located in proximity to sensitive structures due to the heat sink effect and the potential of thermal damage. Irreversible electroporation (IRE) is a non-thermal ablative modality that can deliver targeted treatment and the effective destruction of tumors that are in close proximity to or even surrounding vascular or biliary ducts with minimal damage to these structures. IRE produces short pulses of high-frequency energy which opens pores in the lipid bilayer of cells leading to apoptosis and cell death. IRE has been utilized clinically for over a decade in the treatment of liver cancers with multiple studies documenting an acceptable safety profile and high efficacy rates.

## 1. Introduction

Primary liver cancer is the sixth most common cancer and the third leading cause of cancer death worldwide with over 900,000 new cases and 830,000 deaths during 2020 [1]. Hepatocellular carcinoma (HCC) is the most common primary form of liver cancer, accounting for 75% to 85% of patients, followed by intrahepatic cholangiocarcinoma (10–15%); with other rare types of cancers representing the remainder of cases [1]. In addition to the above, colorectal cancer primarily metastasizes to the liver with 20% to 35% of patients presenting with colorectal cancer liver metastases (CRCLM) at the time of diagnosis and 50 to 70% developing metastasis during the course of their disease [2,3,4].

Complete elimination of the tumor through surgical resection is the recommended approach for the treatment of liver cancers since this offers the best overall survival outcomes [5]. Unfortunately, 70% to 80% of patients with liver cancer are ineligible for surgical resection due to comorbidities, extrahepatic disease, the number of metastases, the location, or size of the tumors [6,7]. While systemic chemotherapy is an option, its use is typically limited to palliative or bridge treatment in patients who are not candidates for surgery or ablation [8,9].

The complex vascular and biliary anatomy of the liver and the prevalence of underlying risk factors, such as cirrhosis, exclude most patients as candidates for surgery [7]. This has led to the development of ablative approaches, such as radiofrequency ablation (RFA), microwave ablation (MWA), cryoablation (CA), and highly focused ultrasound (HIFU), which are designed to destroy tumor cells (Table 1) [10,11]. RFA produces tissue destruction by passing a high-frequency alternating current through the target lesion. MWA uses alternating electromagnetic fields and dielectric heating of water molecules to cause tissue coagulation and is less susceptible to variations from heat sink effect and tissue impedance compared to RFA. HIFU focuses ultrasound energy on the lesions of interest and induces tumor coagulative necrosis as a result of a thermal effect. The goal of ablative techniques is the complete elimination of tumor cells with an adequate margin while minimizing damage to healthy hepatic tissue along with vascular and ductal structures.

While efficacious in the localized treatment of tumors, thermal ablation is contraindicated when target lesions are in proximity to vascular or biliary structures [12]. Extremely high or low temperatures can damage biliary and smaller vascular structures, while larger vascular structures may create a heat sink effect, limiting the efficacy of treatment in their immediate vicinity. All thermal ablation techniques involve a transition zone in which the tissue temperature is not extreme enough to destroy cells but may be high or low enough to cause damage. The temperature gradient from the zone of application of energy to non-affected tissue is influenced by blood flow, tissue composition, tissue density, and other factors; therefore, it is challenging to plan or map with precision [13]. To ensure destruction of the tumor cells with an adequate margin of safety, some amount of healthy liver tissue is sacrificed [14].

The risk of causing complications to adjacent or surrounding vascular or ductal structures when applying thermal energy for the treatment of liver cancer has led to the exploration of irreversible electroporation (IRE) as a non-thermal ablative treatment option. This review aims to provide background information and review the data associated with the use of IRE to treat tumors associated with HCC, CRCLM, and cholangiocarcinoma.

Our narrative review will give a comprehensive overview on the use of irreversible electroporation (IRE) in hepatobiliary malignancies. This review will focus on technical overview of the procedure, patient selection criteria, procedural considerations, safety and complications, clinical outcomes, post-procedural immunological responses, challenges for a new center to starting use of IRE, post-procedural pathological-radiological aspects, and future directions for IRE in the treatment of liver cancers. We will also compare IRE with thermal ablation methods in terms of safety, efficacy, and post-procedural outcomes.

## 2. Technical Overview and Mechanistic Explanation of Irreversible Electroporation

**Figure 1:**
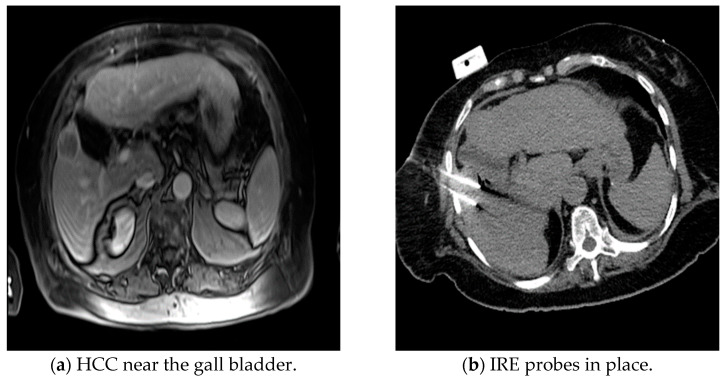

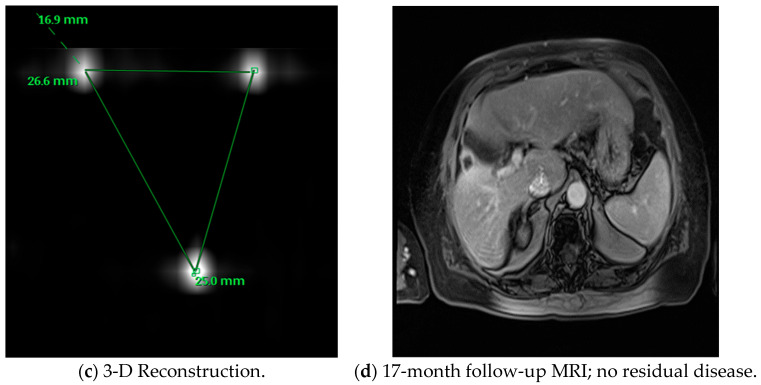
Example of patient with a history of hepatocellular carcinoma treated with IRE.

Electroporation of cells can either be reversible, in the case of chemotherapeutics in electrochemotherapy or nucleic acids in gene electrotransfer, or irreversible, in the case of IRE achieving an ablative effect through the induction of targeted tumor cell death (Figure 1). IRE is a non-thermal ablative technology that delivers low-energy, millisecond pulses of high-voltage direct electrical current to tumor cells which disrupts homeostasis, triggers apoptosis, and ultimately leads to controlled cell death [15].

**Figure 2:**
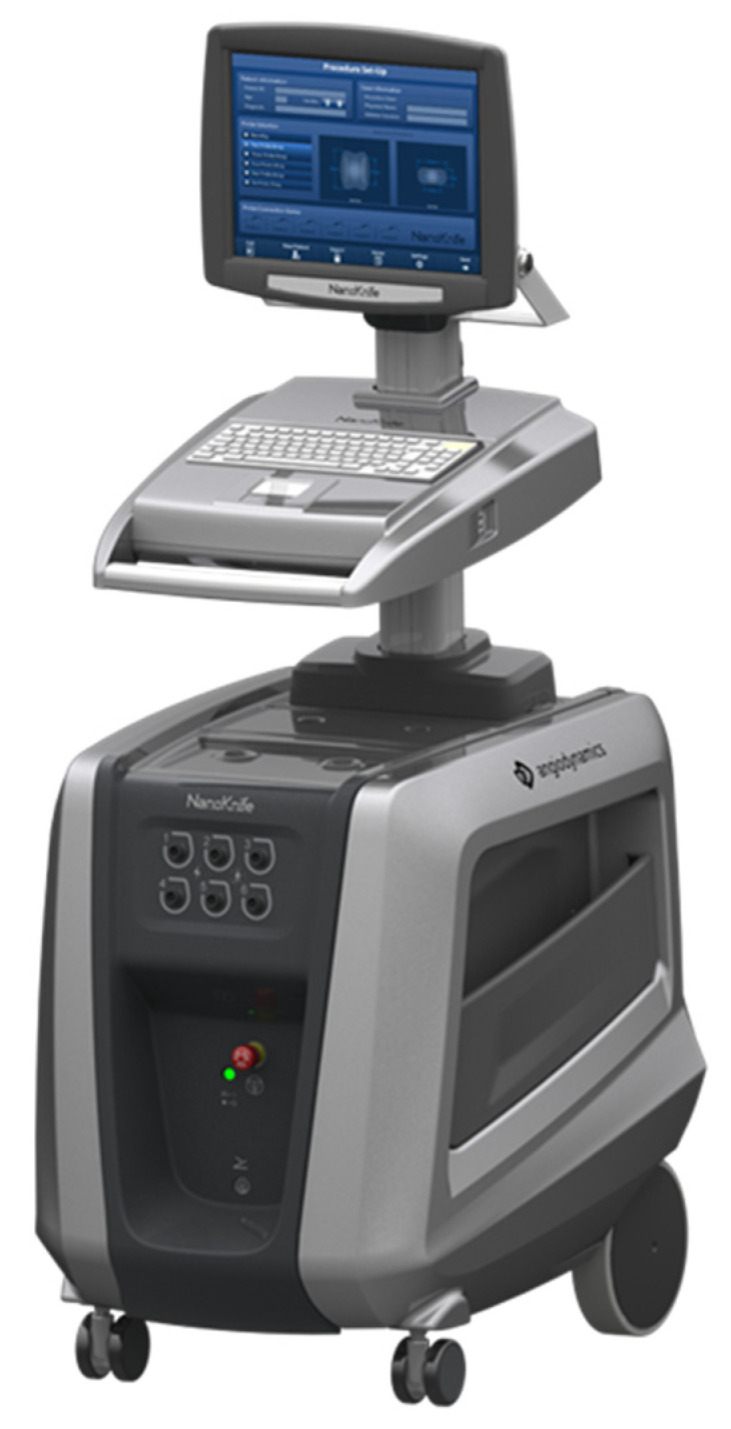
Irreversible Electroporation Generator.

**Figure 3:**
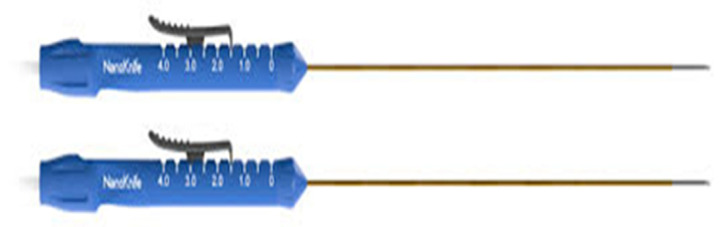
Irreversible Electroporation Monopolar Probes.

**Figure 4:**
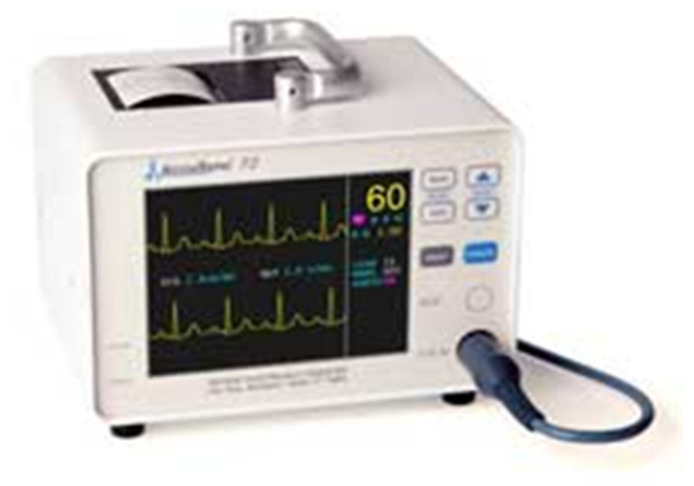
Irreversible Electroporation Accusync Device.

The commercially available IRE device, Nanoknife (3.0 generation, Queensbury, NY, USA), (Angiodynamics, Latham, NY, USA), is composed of three parts. The generator (Figure 2) component produces low-voltage, high-energy DC (direct current) through monopolar probes that are connected to the generator. A minimum of 2 and up to 6 probes are used, with the DC delivered between 2 probes at a time. The second component, the 19-gauge needle monopolar probes (Figure 3), is available in 15 and 25 cm lengths. The active tip of the probe is exposed between 1 and 3 cm and is echogenic to help the operator with visibility during the ablation procedure. Upon insertion, probes must be parallel to avoid convergence or divergence, with ideal spacing between 1.5 and 2.2 cm to obtain the largest ablation volume and avoid high current errors or untreated areas. The third component is the Accusync device (Figure 4). The Accusync is a five-lead system with a printer that aids in synchronization of each pulse delivery with a patient’s electrocardiogram. The Accusync reduces the chance of a cardiac event by detecting the R-wave on the ECG and sending a signal to the generator, which then delays the delivery of a pulse by 0.05 s [16].

Unlike thermal ablative technologies, IRE can be safely performed adjacent to critical vessels and biliary structures, with the area of tissue ablation produced being well defined and lacking in a typical transition zone which is associated with thermal ablation modalities. A cell reaching the irreversible poration threshold will experience cell death while cells which do not reach this threshold will only experience transient opening of the pores [17].

## 3. Patient Selection

IRE can be used in patients with primary or metastatic hepatic malignancies when a non-thermal alternative to thermal ablative technique would be preferred due to a tumor being located near, or surrounding, major vessels or ductal structures. IRE provides an option in these patients to produce complete ablation and avoid the heat sink effect. IRE should be limited to patients with an Eastern Cooperative Oncology Group (ECOG) performance status of 0–2 and an American Society of Anesthesiologists score of 0–3 [18]. Some studies suggest that clinical outcomes are best for lesions ≤3 cm with larger liver tumors being more difficult to treat [19,20]. A recent consensus panel recommended limiting the use of IRE to experienced interventionalists and for lesions less than 5 cm in size [18]. In line with the Society of Interventional Radiology’s consensus guidelines, patients should have an INR of ≤1.5 to 1.8 and platelet levels of greater than 50 × 10^9^/L, general guidelines that have been recommended by the Society of Interventional Radiology [21]. If coagulopathy is not correctable, IRE should not be performed [22].

IRE is contraindicated in patients with a history of cardiac arrhythmias since the electrical pulses it produces cannot be synchronized with the cardiac R-waves, leading to an increase in the risk for ventricular arrhythmias. Early experience with IRE was associated with arrhythmias and cardiac toxicity which led to the development of cardiac synchronization, which is a standard part of the treatment procedure [23]. The incidence of ventricular arrhythmia in conjunction with IRE is rare with a reported range from 0 to 2.5% [24]. Patients with cardiac resynchronization therapy (CRT) devices should also not be treated with IRE.

While cases of seizures associated with IRE treatment have not been reported, epilepsy and a history of seizures are also contraindications for use of IRE since it is speculated that its electrical pulses could potentially provoke a seizure [25]. Additional contraindications include patients with anatomical obstacles which impair the trajectory of IRE electrode insertion, such as an overlying colon or varices in the path to the lesion. Prescreening imaging should be evaluated to identify this risk. Table 2 summarizes the indications and contraindications for the use of IRE for hepatobiliary cancers.

## 4. Procedural Considerations

The IRE procedure is performed under computed tomography (CT) or ultrasound guidance with general anesthesia. IRE has unique anesthesia needs, including preparation for possible arrhythmias, hemodynamic swings, and the need for complete muscular relaxation. Cardiac monitoring is required for electrical synchronization of the ablation pulses with the cardiac cycle. Defibrillator pads should be available due to the potential risk of arrhythmias and cardiac arrest due to electroporation. Propofol and Remifentanil have been used to address increased blood pressure and heart rate which can potentially occur during IRE [26]. Complete muscular relaxation is required during IRE since the delivery of the electrical pulse can lead to generalized muscle contraction [25]. Even with complete paralysis, IRE triggers local contractions of abdominal wall muscles. It is important that anesthesia confirms that there is full neuromuscular relaxation before any electrical pulses are delivered.

The placement of IRE probes requires significant technical expertise and experience with image-guided ablation since the precise placement of the electrodes is essential and often requires insertion in anatomically challenging locations. The number of electrodes required depends on tumor size with up to six probes placed using imaging guidance CT, ultrasound, or a combination of both. CT fluoroscopy enables real-time visualization of the needles, the target lesion, and the surrounding structures during probe placement. The probes should bracket or encircle the entire target lesion with an interprobe distance of no more than 2.2 cm in order to prevent incomplete ablation. For a 2 cm tumor, a triangular array of three electrodes placed at or near the outer edges of the tumor is recommended. The electrodes should also be placed in parallel and not deviate by more than 10 degrees with electrode tips all on the same plane [27]. Misplacing the electrodes by just a few millimeters may lead to incomplete tumor ablation.

Since the ablation zone extends 5 mm outward from the probes, the electrodes should be placed at the edge or just outside of the tumor to achieve a 5 mm margin. For liver tumors, the active tip should be exposed 2.0 cm to prevent under- or overcurrent. If the tumor is larger than 2.0 cm along the access trajectory, repeat ablations can be performed by pulling the electrodes back in 0.5 to 1 cm increments to treat the more superficial area of the tumor [27]. The active tip can be exposed to more than 2.0 cm and up to 3 cm depending on the size and location of the tumor treated and the desired margin.

Ten test pulses are initially delivered between each electrode pair with a target initial current level of 20 to 50 Amperes. The current level is then increased by 12 to 15 Amperes from baseline for the remaining 70 pulses with a typical pulse length of 70 or 90 μs [26]. Intraoperative CT can be used to visualize the gaseous hypodense ablation zone which is produced at the electrode tips as a result of the electrolysis of water into oxygen and hydrogen [28]. The shape and size of the ablation zone on CT are accurate predictors of the treatment zone [29].

Post-procedural management is focused on pain control and monitoring of liver function. Transient rapid elevation of AST, ALT, and bilirubin typically occurs within 24 h of these procedures and resolves spontaneously. The cause of this elevation is not known but is hypothesized to be due to the release of cellular contents after electroporation. On rare occasions, a gradual rise in bilirubin indicating cholestasis may occur which can take days or weeks to normalize [29].

## 5. Safety and Complications

The main advantage of IRE is its use in the treatment of tumors in anatomic locations that are unsafe for thermal ablation approaches. This includes treating tumors abutting or surrounding important vessels. While thermal ablation treats everything within or near the ablation zone, IRE selectively targets cellular walls, killing vascular mesenchymal cells but enabling the wall’s collagen and elastin structures to remain. Since acellular elements within the IRE treatment field such as the extracellular collagen matrix in vessel walls are spared, parenchymal architecture remains intact, enabling re-epithelialization and preservation of vessel and duct function [30,31,32]. Multiple clinical studies have validated the safety of using IRE near blood vessels in the liver. Narayanan et al. observed vascular changes in only 4.4% of 158 vessels after IRE ablation of hepatic tumors with 50 tumors abutting and 10 tumors surrounding vessels [30]. Distelmaier et al. reported no occlusion or narrowing of vessels following the treatment of 43 tumors which were adjacent to major hepatic or portal veins [31]. Tamura et al. demonstrated that while hepatic or portal veins did occlude after IRE, these occlusions were subclinical without ramifications with most occluded veins being less than 4 mm. No hepatic veins greater than 4 mm became occluded, including 14 veins which were within the treatment zone for IRE [33].

IRE can also be safely utilized to ablate tumors within 1.0 cm of bile ducts, gallbladder, and the bowel [32,34,35]. Dollinger et al. reported the successful ablation of 53 tumors adjacent to 55 major bile ducts. Of these, 14 of the tumors directly abutted and 33 surrounded bile ducts. While biliary ductal changes, including mild stenosis or dilatation, were observed on imaging in 15 out of 55 ducts, only three patients developed transient cholestasis that resolved without intervention [32].

Postoperative complication rates after IRE treatment for the treatment of liver cancer are comparable to that of RFA and MWA [26,36]. A systematic review by Scheffer et al. of 16 studies with 129 IRE treated liver tumors reported a 16% complication rate with all reported complications reported being minor (Grade I and II) [26]. Complications were primarily related to probe placement with a higher risk of complications associated with the need for placement of an increased number of electrodes. These included probe-related punctures such as hemothorax, pneumothorax, and pleural effusions. Only three cases of biliary obstructions were reported in these studies with two of the obstructions being a result of local tumor progression as opposed to ablation-induced biliary stenosis.

A retrospective study of 174 procedures in 124 patients in Froud et al. examined changes in liver functions after IRE [29]. Changes in liver function were measured before and after IRE and were followed over a period of time to see if liver functions returned to normal. Bilirubin, aspartate aminotransferase, alanine aminotransferase, and alkaline phosphatase level were measured, with transaminase increasing to extreme levels, 20 times the upper limit of normal in 33% of cases, within 24 h of IRE in 129 cases. Of these cases, 95% returned to normal with a mean of 10 weeks. Both alkaline phosphatase and bilirubin were slower to increase and less likely to return to normal. Alkaline phosphate elevation was seen in 17 out of 174 cases, and resolution was seen in 5 out of the 17 cases. Bilirubin levels were elevated after IRE in 25 out of 174 cases and returned to normal levels in 18 out of the 25 cases. It was concluded that in most post-IRE cases, abnormalities in liver functions resolved without intervention, did not prevent treatment, and showed similar results to those found after RFA or cryoablation [29].

More recently, a large single-center retrospective analysis of 85 IRE ablations of 114 liver tumors reported a 7.1% major (Grade III and IV) and 18.8% minor (Grade I and II) complication rate [37]. The most common major complication was liver abscess which occurred in 4.7% of patients, requiring intravenous antimicrobial and drainage therapy, with the incidence associated with the presence of bilioenteric anastomosis, a known risk factor for liver abscess after ablation or transarterial embolization. One patient with a liver abscess also developed renal failure which was treated with transient hemodialysis. Another patient had an injury to the right mammary artery followed by an acute hemorrhage treated with arterial embolization. Careful analysis of imaging and identification of clinical symptomatology to distinguish normal changes from IRE from abscess formation is needed due to the fact that localized gas pockets can develop in up to 75% of patients treated with IRE, and the appearance of these gas pockets is similar to that of hepatic abscesses.

While IRE treatment requires more probes per treatment than RFA and MWA, reports of treatment-related bleeding are low. There were no bleeding complications reported for any of the 16 studies reviewed by Scheffer et al. [26]. Another large retrospective analysis identified two cases of bleeding requiring transfusion or embolization following the use of IRE for the treatment of 114 liver tumors [37]. A separate retrospective analysis of 43 patients treated with IRE for hepatic tumors identified two cases of subcapsular hematoma and one case of arterioportal fistula [31]. Table 3 offers a comprehensive summary of each of these studies outlining safety and complications.

## 6. Clinical Outcomes

Most studies focusing on the use of IRE to treat primary and metastatic hepatic malignancies have been smaller prospective or retrospective observational studies consisting of combined populations of patients diagnosed with HCC, CRCLM, and cholangiocarcinoma. Niessen et al. retrospectively assessed outcomes associated with the use of IRE for 71 patients with primary liver tumors (HCC and cholangiocarcinoma) or liver metastases [38]. A total of 103 tumors with a median diameter of 1.9 cm (range 0.4 to 4.5 cm) were treated. Median overall survival (OS) was 26.3 months with no difference in median OS between patients with primary and metastatic disease (26.8 vs. 19.9 months; *p* = 0.41). Patients with a tumor diameter >3 cm (*p* < 0.001) or more than two lesions (*p* < 0.005) had a lower overall median OS.

Stillstrom et al. published results from a retrospective review of 42 patients with 59 liver tumors treated with IRE that were not resectable or able to be treated by thermal ablation [39]. Of these tumors, 51% were colorectal cancer liver metastasis (CRCLM) and 34% were hepatocellular carcinomas (HCC). There was no local recurrence reported within 12 months for 61% of the patients. Local recurrence rates throughout the entire group were 26% at six months and 37% at one year. Local recurrence for the CRCLM and HCC groups at one year was 38% and 17% respectively.

Cannon et al. published results from a prospective registry of 44 patients with HCC, colorectal metastases, or other metastases undergoing 48 IRE procedures [20]. Tumors local in proximity to vital structures represent 42% of the lesions recorded. Successful complete ablation was achieved for 100% of the procedures. Overall local recurrence-free survival (LRFS) at six months was 94.6% and 59.5% at 12 months. A trend towards higher recurrence rates was seen for tumors greater than 4 cm in size (HR 3.236, 95% CI: 0.585–17.891; *p* = 0.178).

Frühling et al. conducted a non-randomized single-center study of 30 patients diagnosed with HCC, CRCLM, or other metastases with one or two tumors when surgery, RFA or MWA were not options and tumor size was less than 3 cm (median tumor size was 2.4 cm) [40]. Of these patients, 18 had previously undergone liver resection surgery and 20 had previously received either RFA or MWA. Ablation success at three and six months, defined as no evidence of residual tumor in the ablated area as confirmed by contrast enhanced ultrasound or CT, was 78.9% and 65.8%, respectively. Local recurrence for patients with CRCLM was 26.1% at three months and 47.8% at six months and 28.6% for both the same time periods in patients with metastases due to malignant melanoma or cholangiocarcinoma. There were no local recurrences at either time period for patients with HCC (*p* = 0.084 vs. CRCLM).

More recently, Mafeld et al. reported on a retrospective series of 52 patients with primary hepatic malignancy due to hepatocellular carcinoma, cholangiocarcinoma, or secondary metastatic disease treated with IRE [41]. A complete ablation was achieved in 75% of cases with a median time to progression of eight months. At 12 months, 44% were progression-free (95% CI 30–66%). The authors reported that lesions larger than 2 cm were associated with shorter time to progression and that patients with CRCLM had a more rapid time to progression compared to patients with HCC. Median OS was 38 months with a 90% (95% CI: 72%, 97%) patient survival at 12 months and 65% (95% CI: 40%, 81%) survival at 24 months and 52% (95% CI 22%, 75%) survival at 36 months.

In a patient series limited to individuals diagnosed with HCC, Sutter et al. reported on the use of IRE in 58 patients with 75 tumor nodules [42]. The majority of patients (77.3%) achieved complete tumor ablation after a single IRE procedure with 92.0% tumor ablation after three procedures. Overall local tumor progression free survival (PFS) at six and 12 months was 87% (95% CI: 77%, 93%) and 70% (95% CI: 56%, 81%), respectively.

Hosein et al. reported on a series limited to patients with CRCLM who received percutaneous IRE ablation [35]. A total of 29 patients with 58 tumors having a median tumor size of 2.7 cm were treated. Grade 1 abdominal pain was reported by most patients, and no procedural deaths were reported. At two years following the procedure, median OS was 62% (95% CI: 37%, 87%) and median PFS 18% (95% CI: 0%, 35%). Of these patients, 36% experienced a complete response, 21% a partial response, 25% stable disease, and 18% progressive disease at a median follow up of 11 months.

While the prevalence of cholangiocarcinoma is less than it is with other liver tumors, several authors have published on the safety and efficacy of IRE specifically in this patient population [43,44]. This includes a retrospective study by Martin et al. of 26 patients with obstructive jaundice related to advanced hilar cholangiocarcinoma (AHC) who underwent IRE ablation of the tumor [44]. Patients were compared to a control group of 137 patients receiving standard of care only consisting of percutaneous transhepatic biliary drainage (PTBD) with no ablation. Median time to PTBD removal was 122 days (range 0 to 305 days) for the IRE group with a median catheter-free time before requiring PTBD replacement of 305 days (range 92–458 days). The control cohort experienced a 59% admission rate for the treatment of PTBD-related infection, occlusion, or other catheter-related problems. The authors concluded that the use of IRE provided effective relief of symptoms and reduced dependency on PTBD.

Another prospective study by Franken et al. reported on 12 patients with perihilar cholangiocarcinoma (PHC) treated with IRE in a multicenter, Phase I/II safety and feasibility study [45]. Patients that were included had locally advanced PHC with vascular, N2 lymph node involvement, or local recurrent PHC at the hepaticojejunostomy. Of these patients, six had major adverse events (CTCAE grade ≥ 3) and no 90-day mortality was reported. Technical success was reported in 100% of the cases. No intraprocedural events related to IRE occurred. The authors concluded that percutaneous CT-guided IRE ablation was relatively safe and feasible. Table 4 offers a comprehensive summary of studies that examine various clincal outcomes.

While the above is not a complete synopsis of all studies and experiences reported to date with IRE for patients with primary and metastatic hepatic malignancies, they provide substantial evidence that the use of IRE provides a safe and effective alternative for the complete ablation of liver tumors in patients with contraindications to other commonly used ablative techniques. Additionally, unlike RFA and MWA, the efficacy of IRE is not influenced by the heat sink or thermal cooling effect from adjacent circulating blood since it has a non-thermal mechanism of action and can be used in the treatment of tumors directly abutting or surrounding large vascular structures such as portal veins, hepatic arteries, or hemangiomas.

Patients with non-metastatic liver disease, smaller tumor sizes, and a smaller number of lesions typically have more beneficial outcomes with IRE. The increased effectiveness of IRE in patients with smaller tumors is expected since the procedure is technically demanding and requires multiple electrodes to be precisely placed to achieve complete ablation. As experience with IRE has increased, there has been increased success with the ablation of larger tumors that has led to a consensus recommendation that IRE can be used in patients with liver lesions up to 5 cm [18].

## 7. Post-IRE Immunological Responses

After ablative procedures, cell debris from the ablated tumor can act as a source of tumor antigens. The cell debris, along with danger-associated molecular patterns (DAMPs) released from damaged or dying cells, can induce an immune response after the ablation. The immune responses that are induced by RFA and MWA ablation, however, are typically short lived, with RFA showing an 87.5% decrease in cytotoxic T cells shortly after ablation, and MWA showing a drop in T cells 72 h after ablation. It has been speculated that these short-lived T cell responses could be due to the thermal ablative techniques causing the denaturation of any released tumor antigens, further decreasing the release of inflammatory DAMPs [46].

Dai et al. utilized an animal model to analyze the immunological effects of IRE on hepatocellular carcinoma (HCC) [46]. In this study, 74 mice were used in four separate animal models. First, 16 mice with HCC were split into two groups: HCC and HCC + IRE. The eight IRE-treated mice along with an additional eight normal age-matched mice were given H22 cells, a cell line derived from a mouse hepatoma; 30 days after the tumor cell injection, Splenocytes were isolated. The Splenocytes were used to detect IFN-y ^+^, CD4 ^+^, and CD8 ^+^ T cells. In the second animal model, 16 HCC mice were randomly given IRE or no treatment, and Splenocytes plus tumor tissue were isolated after seven days. In the third model, nine tumor-bearing mice were split into control, IRE + antiCD8 (blocking antibody), and IRE groups. Tumor volume, weight, metastasis, and Splenocytes (IFN-y^+^ and CD8^+^) were analyzed after 21 days. In the fourth model, 15 mice were injected with tumor lysates from IRE-treated H22 cells in one flank. After 30 days, these 15 mice, along with 10 normal age-matched mice, were injected with H22 cell lysates in the opposite flank, and Splenocytes (IFN-y^+^, CD8^+^, dendritic cells) were analyzed after 21 days.

Each of these mouse models led the authors to six major conclusions: IRE contributed to local tumor regression and a systemic antitumor immune response, IRE increased CD8 ^+^ T cell infiltration in tumor tissue and the spleen, IRE induced long-lasting immunity with a dependence on CD8 ^+^ T cells to prevent regrowth and metastasis, IRE played a major role within the systemic immunosuppressive environment, and IRE led to necrosis and the release of DAMPs. Overall, the authors concluded that IRE had a direct role in the induction of a systemic antitumor response with long-lasting protection [46].

A separate prospective study compared the systemic immune responses in HCC patients after treatment with IRE versus RFA [47]. Of the 21 patients included, 11 were treated with RFA and 10 were treated with IRE. Blood samples were collected before ablation, within 1 h after ablation, 24 h after ablation, and 4 days after ablation. Plasma and peripheral blood mononuclear cells (i.e., lymphocytes, monocytes, and dendritic cells) were collected from blood samples at the four different timepoints. Plasma levels of selected chemokines and cytokines were also collected. The ablation zone for both RFA and IRE was assessed one day after procedure with CT. Follow-up scans included MRI or CT at three-month intervals to evaluate local tumor progression, defined as enhancing tumor within 1 cm of the ablation zone [47].

The results of this prospective study determined that IRE could be clinically beneficial due to the minimal damage that it causes to the liver and the comparatively quick reparative process that was observed in the follow-up MRI/CT imaging. CD14+ monocytes, a type of white blood cell involved in immune response and inflammation, were also found to quickly accumulate in the post-IRE ablation zones. A 9.3-fold increase was observed in Macrophage migration Inhibitory Factor (MIF) levels, 1-h post-IRE. This is an immunostimulatory cytokine responsible for an inflammatory response and was found to quickly return to normal after one day, while with RFA MIF levels had a 1.8-fold increase and did not return to normal until four days post-ablation. The authors speculate that the rise and fall of MIF levels may hold a significant role in the post-IRE immunological process. The post-IRE inflammatory response releases MIF into the ablation zone where it exhibits protective effects and ultimately aids in the repopulation of hepatocytes. MIF also recruits macrophages, monocytes, and leukocytes from the systemic circulation to the ablation zone, and this may aid in an earlier start to the post-ablative healing process. IRE also induces less liver fibrosis in comparison to RFA, which could be due to the difference in MIF levels. No significant difference in IL-6 levels was seen in IRE versus RF ablation. The study concluded that early increases in the MIF levels were seen in IRE, leading to an overall earlier reparative process [47].

## 8. Challenges for New Centers to Start Using IRE

IRE as an ablative modality poses a unique set of challenges. The technology necessitates the need for general anesthesia and complete muscle relaxation, which requires the anesthesia service to be part of the procedure. Capital and disposable costs are two factors common to all ablation modalities; however, with IRE, at least two probes will always be required, unlike MWA where, most of the time, a single probe might suffice. A learning curve with the technical aspects required for IRE procedures could pose a challenge; however, for an interventional oncologist who regularly performs ablations, the learning curve may be less steep. IRE would be a complement to any interventional oncology program that offers various ablation treatment choices. It complements thermal ablation by offering the ability to treat lesions near vasculature, bile ducts, gallbladder, and other critical structures.

## 9. Post-IRE Pathologic-Radiologic Aspects

The ability of IRE to induce tumor necrosis and cell death can be evaluated through varying imaging and pathological methods. Beicos et al. evaluated the clinical efficacy of IRE through the histologic and imaging responses of hepatic and pancreatic cancer that were resected after IRE treatment [48]. This retrospective review of a prospectively collected database examined 11 patients with 12 lesions treated with IRE. Of these lesions, three were pancreatic carcinomas, five were primary liver lesions, and four were metastatic liver tumors. The mean lesion size was 2.8 cm and the mean ablation zone size was 4.4 cm. Using histological evaluation post-IRE, complete response (CR) was seen in three lesions, partial response (PR) was seen in eight, and no response (NR) was seen in one. None of the lesions showed vascular thrombosis or luminal obliteration, and the histologic findings showed biliary duct preservation in each ablation zone. Of the nine liver lesions, two had CR, six had PR, and one had NR.

A second retrospective study examined both radiologic and pathologic findings post-IRE treatment. Cheng et al. evaluated six patients who underwent IRE between 2011–2013, followed by a liver transplant, for HCC [49]. Follow-up imaging was completed at both one and three months following liver transplant, and the assessment of imaging responses was based on modified RECIST criteria. The results showed that all the tumors treated with IRE displayed complete response on all the one- and three-month follow-up imaging and none of the tumors required additional treatment between IRE and transplant. The authors’ pathologic findings found that five tumors, measuring between 12–30 mm, demonstrated complete cell death and all treatment zones displayed confluent necrosis. Nonviable HCC was seen with the use of higher magnification. Overall, the authors determined through both radiologic and pathologic findings, IRE induced cell death and preserved bile ducts and vessels within the treatment areas.

## 10. Comparison between IRE and Thermal Ablation Methods

Bhutiani et al. compared IRE to MWA in a series of 55 Child-Pugh B patients with HCC who were not candidates for transplant or resection or were receiving treatment as a bridge to transplant or had a single remaining lesion after drug-eluting bead therapy [50]. Outcomes accessed included ablation success, tumor recurrence, morbidity, and survival. Treatment success was similar between the two groups with 97% of IRE patients and 100% of MW patients (*p* = 0.37) achieving complete ablation of their liver tumors. Patients undergoing IRE experienced a rate of lower major and minor complications (IRE 27% vs. MWA 76%), had shorter median length of stay (*p* = 0.05) and lower 90-day readmission rate (IRE 13% vs. MWA 36%; *p* = 0.03) than those undergoing MWA.

Liu et al. compared the safety, efficacy, and intermediate-term outcomes of both RFA and IRE in the treatment of liver lesions [51]. The prospective, double-arm clinical trial included 24 patients with 27 lesions with either primary or secondary liver lesions. The groups were randomized, efficacy was measured as local ablation control evaluation at 90-days, safety was measured as procedure-related complications at ≤90 days, and intermediate-term survival was measured at 24 months. In this study, 10 lesions were treated with IRE and 14 lesions treated with RFA. The overall complication rate was 58.3% and the RFA group had a higher number of grade II complications compared to the IRE group. The IRE group had 1 grade IV-b and 1 grade V complication. A total of 23 patients were able to complete follow-ups for the study and 13 survived for 24 months after the procedures. The average OS was 18.17 months; the OS for the IRE group was 17.55 months and for the RFA group 18.75 months. There was no statistical difference in the OS for the RFA compared to the IRE group. The study concluded that IRE is both safe and effective, does less damage to critical structures or vessels surrounding targeted lesions compared to thermal ablative techniques, and was shown to have similar safety, efficacy, and OS profiles as RFA.

Wada et al., a single-center retrospective study, compared safety and treatment outcomes in RFA, IRE, and MW ablation in the treatment of early-stage HCC [52]. Between January 2018 and October 2021, patients with HCC treated with RFA or MWA were identified. Between January 2014 and October 2021, patients with HCC treated with IRE were identified. A total of 322 patients with 366 HCC lesions were treated. Of these patients, 15 were treated with IRE, 216 were treated with RFA, and 91 were treated with MW ablation. Overall, the authors concluded that IRE showed significantly better local tumor control than RFA in both a propensity score-matched and unmatched cohort. The authors also concluded IRE should be used on lesions that are adjacent to vascular structures to prevent any local tumor progression (LTP). On the other hand, there was no significant difference in local tumor control when either MWA or IRE was used in both unmatched and propensity-matched groups. There were also no noted differences in terms of the frequency and severity of complications for both MWA or RFA. There was no significant difference in the two-year recurrence free survival rate between RFA, IRE, and MWA. Overall, the authors determined that IRE over RFA offers better local tumor control for small perivascular HCC lesions, and RFA and MWA display similar outcomes in early-stage HCC.

## 11. Future Directions with IRE for Liver Cancers

Continued research and development efforts with IRE have focused on the underlying mechanisms in which IRE influences the course of disease, the potential for combining IRE with immunomodulatory therapies, and newer techniques for delivering IRE therapy. Research efforts have focused on gaining a greater understanding of post-IRE alterations in the tumor microenvironment using staining techniques and transmission electron microscopy [53]. Having a better understanding of these effects at the cellular level may lead to the identification of in vivo biomarkers which can provide noninvasive detection of tumor response. In addition to the study of these local effects, gaining a better understanding of systemic effects can also provide further insights on the potential for IRE therapy.

Animal studies have demonstrated that irreversible electroporation can cause the release of tumor specific antigens to the tumor microenvironment, elicit an immune response, and evoke an infiltration of macrophages and T cells [54,55]. The above may produce an abscopal effect which occurs when local tumor treatment not only shrinks the targeted tumor but also leads to the shrinkage of untreated tumors located elsewhere.

High-frequency irreversible electroporation (HFIRE) is a technique in development that may eliminate the need for intraoperative paralytics and cardiac synchronization in conjunction with IRE therapy [56]. HFIRE uses bipolar square waves of 1–5 μs in rapid bursts to reduce the risks for cardiac asynchrony and muscle tetany. HFIRE can also be performed with a single-needle, dual-electrode device which reduces the current need for a high level of technical skill required to accurately place and align multiple IRE electrodes in order to obtain optimal treatment results.

Results from a preclinical feasibility canine study of single-needle, high-frequency irreversible electroporation (SN-HFIRE) for the treatment of HCC were reported [56]. In this study, three canines with resectable HCC were treated with SN-HFIRE without cardiac synchronization or intraoperative paralytics. Three hundred 2250 V bursts with 2 μs pulse widths and a 5 μs delay between each change in polarity were used for a total on-time of 100 μs for each burst. Adverse events, SN-HFIRE-induced local immune response, and ablation volumes measured using post-treatment CT were evaluated. Treatment-specific lethal thresholds for malignant and healthy liver tissue were also determined. SN-HFIRE treatment produced predictable ablation volumes (mean volume of 3.89 cm^3^ ± 0.74) as assessed by post-treatment CT with no detectable cardiac interference and minimal muscle contraction. No adverse events were reported. Immunohistochemical analysis demonstrated a well-defined ablation zone composed of collagen and CD3+/CD4−/CD8− lymphocytes. While further studies of SN-HFIRE are needed, this exploratory study provides promising results and supports the potential feasibility of this technique. Large studies are needed to determine the potential clinical application of this approach which could increase the utility of IRE and also enable its use in patients with underlying cardiac rhythm abnormalities.

The complexity of liver cancer pathology often requires the participation of multiple medical subspecialities. The integration of interventional radiology within a multidisciplinary approach offers new and advancing treatment options that could improve overall patient outcomes. In centers with a mature and comprehensive ablation program, IRE presents a safe and viable option in treating hepatobiliary malignancies in close proximity to critical structures such as bile ducts, gall bladder, and vasculature. The option of IRE is typically discussed in a multidisciplinary setting like any other interventional oncology treatment option with a recommendation to be used in cases where it will offer an advantage over thermal ablation techniques [57,58].

## 12. Conclusions

Irreversible electroporation is a unique non-thermal ablation modality for primary and metastatic hepatic malignancies. IRE provides a therapeutic option for the effective destruction of tumors which are in close proximity to or surrounding vascular or biliary ducts in patients who are not candidates for surgical resection or treatment with thermal ablation technologies. Numerous studies have demonstrated the safety and effectiveness of IRE in the treatment of tumor lesions in this patient population. Newer research is currently underway to further elucidate the effect of IRE on the tumor microenvironment and identify the potential for IRE to enhance systemic immunomodulatory response. The development of next generation IRE devices which improve ease of use and reduce current limitations associated with the technology are underway.

## Figures and Tables

**Table 1:** Comparison of Ablative Modalities in Hepatobiliary Cancer.

Technology	Mode of Action	Advantages	Limitations
Radiofrequency ablation (RFA)	Thermally induced coagulation necrosis generated by high-frequency alternating current.	Widely available. Relatively inexpensive. Effective.	Potential for inadequate treatment in proximity to large vessels. Risk of thermal damage to adjacent structures. Tissue charring. Larger tumors (>2.5 cm) require multiple electrodes.
Microwave ablation (MWA)	Thermal ablation induced through agitation of water molecules.	Does not require grounding pads. More predictable lesion as compared to RFA. Can treat larger tumors. Fast acting.	Potential for inadequate treatment in proximity to large vessels. Risk of thermal damage to adjacent structures.
Cryoablation	Changes in argon gas pressure generate freeze-thaw cycles. Probe tip temperatures of −185° can be reached.	Relatively lower postoperative pain as compared to RFA and MWA.	Potential for inadequate treatment in proximity to large vessels. Risk of thermal damage to adjacent structures.
High Intensity Focused Ultrasound (HIFU)	Thermal coagulation combined with cavitation.	No percutaneous probes.	Potential for inadequate treatment in proximity to large vessels. Risk of thermal damage to adjacent structures.
Irreversible electroporation (IRE)	Electrical pulses which create permanent pores in cell membranes leading to apoptosis.	Non-thermal. Can be used near vessels and ducts. Preservation of extracellular matrix and parenchymal structures.	Risk of arrhythmia. Requires general anesthesia, muscle relaxation, and cardiac synchronization. Technically challenging and time consuming.

**Table 2:** Indications and Contraindications for the Use of IRE for Hepatobiliary Cancers.

Indications	Relative Contraindication	Absolute Contraindication
Patient		
Oligometastatic disease in poor surgical candidates ECOG of 0–2 ASA of 1–2	Atrial fibrillation Correctable coagulopathy Limited extrahepatic disease ASA of 3	Ventricular arrhythmia Pacemaker or implantable cardioverter defibrillator (ICD) Uncorrectable coagulopathy ASA of >3, ECOG of >2 Prior history of epilepsy or seizures
**Anatomic**		
Near major bile ducts Near bowel Near major vessels or other structures which may lead to significant heat-sink effect Tumors < 3 cm	Bilioenteric sphincter * (needs antibiotics) Superficial lesions Metal stents Tumors 3–5 cm	Intrahepatic bile duct dilation Exophytic tumor due to risk of seeding Tumors > 5 cm

* Increased risk of infection, patients are given antibiotics before and after procedure.

**Table 3:** Synopsis of Studies Used in Safety and Complications.

Study	Patient Characteristics	Tumor Types	IRE Parameters	Major Outcomes
Narayanan et al. [30]	101 patients, ages 24–83, 129 lesions,158 vessels examined for patency on follow-up	Liver (100), Pancreas (18), Kidney (3), Pelvis (1), Aorto-caval lymph nodes (2), Adrenal (2), Lung (1), Retroperitoneal (1), Surgical bed of prior Whipple (1)	90 high-voltage (1500–3000 V) direct current (25–45 A) electrical pulses were delivered, in nine sets of 10 pulses between paired unipolar electrodes or a single bipolar electrode.	Vascular changes in 4.4% (7/158) after IRE of hepatic tumors with 50 tumors abutting and 10 surrounding vessels
Distelmaier et al. [31]	29 patients, mean age 63 years ± 12	8 primary, 35 secondary malignant liver tumors located immediately adjacent to major hepatic veins, portal veins or both	70–90 pulses per probe pair, pulse length 90 μs, max voltage 3000 V w/electrocardiographic triggering	No occlusion or narrowing of vessels post-IRE procedure for 43 hepatic tumors
Tamura et al. [33]	39 patients, mean age 57.8 years ± 11.8	Colon (27), Intrahepatic (2), HCC (1), Hilar (1), Esophageal (2), Pancreatic (1), Mammary (1), Ewing sarcoma (1); Primary (1), Metastatic (38)	70–90 pulses per probe pair; Pulse length 90 μs; Max voltage 3000 V with electrocardiographic triggering	33 portal veins and 64 hepatic veins analyzed; Occlusions of hepatic/portal veins were subclinical w/out ramifications w/most being less than 4 mm; No hepatic veins larger than 4 mm became occluded
Froud et al. [29]	174 ablation procedures in 124 patients, mean age	Liver lesions included metastatic disease (62), with colorectal making up 31/62; Primary liver cancer (62), HCC (53), Cholangiocarcinoma (8) and (1) unknown diagnosis	70–90 pulses per pair using between 1500–3000 V	In most post-IRE cases, abnormalities in liver functions resolved without intervention, did not prevent treatment, and showed similar results to those found after RFA or cryoablation
Dollinger et al. [32]	24 patients, mean age 59.3 years, 53 hepatic lesions in 35 ablation procedures	53 hepatic tumors w/14 primary; Segment IV (20), Segment V (10), Segment VI (1), Segment VII (6), Segment VIII (16)	Two to six monopolar 18-gauge IRE probes were placed parallel to each other in or around the target tumor; 70 pulses per cycle; 90 µs pulse length; Voltage-to-distance ratio, 1500 V/cm of needle distance	Successful ablation of 53 tumors adjacent to 55 major bile ducts; Biliary ductal changes, including mild stenosis or dilatation, were observed on imaging in 15 out of 55 ducts, only 3 patients developed transient cholestasis that resolved without intervention
Scheffer et al. [26]	16 studies, 221 patients with 325 treated tumors	Patients presenting with lesions in liver (129), Pancreas (69), Kidney (14), Lung (6), Lesser pelvis (1), Lymph node (2)	Heterogeneity of reporting details, i.e., interelectrode distance, applied voltage + resulting current, pulse duration, number of electrodes, and probe repositioning, did not allow for detailed review of parameters	16% complication rate (Grade I and II); Higher risk of complications associated with placement of more electrodes, i.e., probe-related punctures such as hemothorax, pneumothorax, and pleural effusions; 3 cases of biliary obstructions with 2 being a result of local tumor progression as opposed to ablation-induced biliary stenosis
Dollinger et al. [37]	85 IRE procedures in 56 patients; Patient group consisted of 42 men and 14 women with a median age of 61 years (range, 22–81 years)	28 patients with 52 lesions of primary liver tumors; HCC (45), CCA (7); 28 patients with 62 lesions of secondary liver tumors; Colorectal tumor (44), Breast carcinoma (6), Neuroendocrine tumor (3), Pancreatic tumor (3), Other (6)	Voltage 1650–3000 V; 90 µs pulse length; 70 pulses per cycle	7.1% (6/58) experienced major complications with hepatic abscess in 4.7% (4 patients), bleeding in 2.4% (2 patients), 1 patient needing arterial embolization and 1 a blood transfusion; Minor complications in 18.8% (16/85), minor hemorrhage in 5.9% (5), portal vein branch thrombosis in 5.9% (5), pneumothorax with no chest drain in 3.5% (3), hepatic arteriovenous shunt in 3.5% (3), and temporary neurologic deficits due to peri-interventional positioning in 2.3% (2)

**Table 4:** Synopsis of Studies Used in Clinical Outcomes.

Study	Patient Characteristics	Tumor Types	Ablation Parameters	Major Outcomes
Niessen et al. [38]	71 patients, median age 63.5 ± 10.8 years	103 liver tumors, 35 patients had primary liver tumors, 36 had liver metastases; 43.7% HCC, 5.6% Cholangiocarcinoma, 38% Colorectal, 12.7% other metastases	1650–3000 V; Pulse length 90 µs; 70 pulses per cycle under constant EKG monitoring	Median overall survival (OS) was 26.3 months, no difference in median OS between patients with primary and metastatic disease (26.8 vs. 19.9 months; *p* = 0.41). Patients with a tumor diameter >3 cm (*p* < 0.001) or more than 2 lesions (*p* < 0.005) had a lower overall median OS
Stillström et al. [39]	42 patients had 50 treatments, 59 tumors	59 tumors, 51% colorectal liver metastases, 34% HCC	10–20 test pulses delivered b/t each electrode pair, minimum of 70 treatment pulses delivered b/t each electrode pair	No local recurrence within 12 months for 61% of the patients; Local recurrence rates for the entire group were 26% at 6 months and 37% at 1 year; Local recurrence for the CRCLM and HCC groups at 1 year was 38% and 17% respectively
Cannon et al. [20]	44 patients, 48 IRE ablations	20 colorectal lesions, 14 HCC, 10 other metastasis	3000 V pulses, 90 pulses delivered lasting 20–100 µs each; Nanoknife system	Overall local recurrence-free survival (LRFS) at 6 months was 94.6% and 59.5% at 12 months; Higher recurrence rates was seen for tumors greater than 4 cm in size (HR 3.236, 95% CI: 0.585–17.891; *p* = 0.178)
Frühling et al. [40]	30 patients with 38 lesions treated with IRE between September 2011 and September 2014; Mean age 63 years	23 CRLM (colorectal cancer w/liver metastasis), 8 HCC, 7 other metastases	Minimum 90 treatment pulses delivered b/t each adequate electrode pair (distance not exceeding 25 mm); Current 40 A, no less than 30 A	Ablation success 78.9% at 3 months, 65.8% at 6 months; 6 minor complications, 1 major complication; No mortality at 30 days
Mafeld et al. [41]	52 patients, 59 lesions, mean age of 64 years (range from 28–94), primary or secondary hepatic malignancy	Primary: HCC (20), Cholangiocarcinoma (3); Secondary: Colorectal (28), Neuroendocrine (1), Pancreatic (1), Breast (1), Gastrointestinal stromal tumor (1), Malignant thymoma (1); Mean diameter 2.4 cm	90 pulses, 1500 v/cm applied b/t each electrode pair (including test pulses); Range of 20–50 A; Electrodes placed in parallel 1–2 cm apart	12 months, 44% were progression-free (95% CI 30–66%); Lesions larger than 2 cm were associated with shorter time to progression and patients with CRCLM had a more rapid time to progression compared to HCC; Median OS was 38 months with a 90% (95% CI: 72%,97%) patient survival at 12 months and 65% (95% CI: 40%,81%) survival at 24 months and 52% (95% CI 22%, 75%) survival at 36 months
Sutter et al. [42]	58 patients, median age 65.4 years, range of 41.6–90 years, 75 HCC lesions	75 HCC tumors, median lesion diameter 24 mm (range of 6–90 mm)	Nanoknife IRE; Max 3000 V and 50 A; 2–6 19-gauge electrodes w/adjustable exposure of length of active tip (5–40 mm); Cardiac synchronization; Electrodes placed parallel w/max distance of 2.5 cm	77.3% achieved complete tumor ablation after single IRE procedure with 92.0% tumor ablation after 3 procedures; 6 and 12-month overall local tumor progression free survival (PFS) was 87% (95% CI: 77%, 93%) and 70% (95% CI: 56%, 81%)
Hosein et al. [35]	29 patients, 58 lesions, 36 IRE procedures, median age of 62 years	58 tumors, median number of lesions treated was 2, median lesion size was 2.7 cm	Nanoknife IRE; 70-ms, 1500–3000 V, 25–45 A	2 years after procedure, median OS was 62% (95% CI: 37%, 87%) and median PFS 18% (95% CI: 0%,35%); 36% of patients had complete response, 21% partial response, 25% stable disease, and 18% progressive disease at median follow up 11 months
Martin et al. [44]	26 patients with obstructive jaundice due to advanced hilar cholangiocarcinoma treated with IRE with median age of 63 years, 137 patients with no ablation (control) with median age of 61 years	Advanced stage 3 or 4 hilar cholangiocarcinoma causing obstructive jaundice, IRE patients had 26 total lesions, 137 non-IRE (control) patients	Goal to perform 100 electrical pulses in groups of 10, pulse duration 70–90 µs, pulse interval of 250 ms	After percutaneous transhepatic biliary drainage (PTBD), 2 patients had ≥grade 3 complications; IRE resulted in relief of biliary obstruction and let patients live w/out PTBD for median 10 months
Franken et al. [45]	12 patients, mean age of 63 years ± 12	Unresectable locally advanced perihilar cholangiocarcinoma or N2 lymph node involvement	Active tip length 1.5–2 cm w/interelectrode distance of 10–24 mm w/5 mm margin around lesion; 90 treatment pulses, 9 sets of 10 pulses b/t paired unipolar electrodes; Voltage setting 1500 V/cm	6 patients had major adverse events (CTCAE grade ≥ 3); No 90-day mortality; Technical success in 100% of cases; No intraprocedural events related to IRE

## Data Availability

No new data were created or analyzed in this study. Data sharing is not applicable to this article.

## Glossary

High-Focused Ultrasound (HIFU)	Non-invasive technique that uses focused ultrasound to induce cell death
Microwave Ablation (MWA)	Thermal ablative technique that uses heat energy generated from microwaves to induce cell death
Irreversible Electroporation (IRE)	Non-thermal ablative technique that utilizes direct current to induce cell death
Cryoablation (CRYO)	Thermal ablative technique that uses
Radiofrequency ablation (RFA)	Thermal ablative technique that uses alternating current to induce cell death
Nanoknife	Commercial name of irreversible electroporation device
